# Identification of urine biomarkers predictive of prolonged QTc interval in multidrug-resistant tuberculosis patients treated with bedaquiline

**DOI:** 10.3389/fphar.2024.1362544

**Published:** 2024-05-30

**Authors:** Jiajia Yu, Weicong Ren, Jinfeng Yuan, Rongmei Liu, Liping Ma, Shenjie Tang, Yu Pang

**Affiliations:** ^1^ Department of Bacteriology and Immunology, Beijing Chest Hospital, Capital Medical University/Beijing Tuberculosis and Thoracic Tumor Research Institute, Beijing, China; ^2^ Department of Tuberculosis, Beijing Chest Hospital, Capital Medical University/Beijing Tuberculosis and Thoracic Tumor Research Institute, Beijing, China; ^3^ Clinical Center on Tuberculosis, Beijing Chest Hospital, Capital Medical University, Beijing Tuberculosis and Thoracic Tumor Research Institute, Beijing, China

**Keywords:** metabolomics, tuberculosis, bedaquiline, urine, biomarkers

## Abstract

The most frequent adverse event associated with bedaquiline (BDQ) is the QTc interval prolongation; however, there was no biomarkers that could be used to predict the occurrence of QTc prolongation in BDQ-treated patients. In this study, we employed the ultra-high performance liquid chromatography-MS/MS (UHPLC-MS/MS) to generate metabolic profiling for the discovery of potential predictive urine biomarkers of QTc prolongation in these patients. Untargeted metabolomic technique was used to concentrate the differential metabolic pathway, and targeted metabolomic technique was subsequently performed to identify predictive biomarkers for QTc prolongation. A total of 45 rifampicin-resistant TB (RR-TB) and multidrug-resistant TB (MDR-TB) patients were enrolled in our study, including 15 RR/MDR-TB patients with QTc interval prolongation (QIP) and 30 RR/MDR-TB patients with QTc interval un-prolongations (QIU). Untargeted technique revealed that the lipid metabolism was the most differential metabolic pathway between two groups. Further targeted technique identified four differential metabolites, including betaine, LPE (18:2), LPE (20:3), and LPE (20:4). The combined analysis of metabolisms revealed that the combined use of LPE (20:3) and LPE (20:4) had the best performance for predicting the occurrence of QTc prolongation in TB patients, yielding a sensitivity of 87.4% and a specificity of 78.5%. In addition, with the progression of BDQ treatment, the LPEs exhibited persistent difference in the BDQ-treated TB patients experiencing QTc interval prolongation. In conclusion, our data demonstrate that the combined use of LPE (20:3) and LPE (20:4) yields promising performance for predicting the occurrence of QTc interval prolongation in BDQ-treated patients.

## Introduction

Tuberculosis (TB), caused by *Mycobacterium tuberculosis* complex, remains a major public health concern in spite of the significant impact of efforts made to improve its control worldwide. The World Health Organization (WHO) estimates that 10.6 million individuals became ill with TB and 1.3 million died in 2022 (World Health Organization, 2023). The emergence and spread of drug-resistant TB, especially rifampicin-resistant TB (RR-TB) and multidrug-resistant TB (MDR-TB), is considered as a significant challenge that hamper effective TB control. RR/MDR-TB cases require longer and more toxic therapy, and usually experience poorer outcomes. The low treatment success rates emphasize a high potential transmission risk of RR/MDR-TB within the community (Dookie et al., 2022). Fortunately, there have been greater efforts in recent years to address the problem of RR/MDR-TB (Nyang’wa et al., 2023). Several new or repurposed antimicrobial drugs are in advanced-stage trial or in late-stage development for RR/MDR-TB, providing new hope for treatment of this disease.

Bedaquiline (BDQ), a new diarylquinoline antibiotic with specific activity against MTB, has been endorsed by the WHO for the treatment of RR/MDR-TB. Indeed, a series of studies demonstrate that BDQ-containing regimens have shown impressive efficacy in the RR/MDR-TB therapy. Despite evidence of safety of BDQ when used in different populations, it has the potential to affect cardiac electrophysiology by prolonging the QTc interval. In a recent Chinese cohort employing BDQ, QTc prolongation was noted in approximate one-fifth patients, which was also the major reason promoting BDQ discontinuation (Gao et al., 2021). Similarly, a systematic analysis of the evidence revealed that QTc longer than 450 ms was reported in 10.6% patients exposed to BDQ (Pontali et al., 2017). It also been suggested that the prolonged QTc potentially associated with BDQ caused 10 unexplained deaths in the BDQ-treated arm of a proprietary trial (Diacon et al., 2014). The WHO advises the strict monitoring procedures when administering this medication (World Health Organization, 2013); however, the routine monitoring could not make the diagnosis in a timely manner, and delay the interruption for severe adverse events. Therefore, there is an urgent need to establish an approach to identify patients at high risk for QTc prolongation.

Urine is a highly desirable specimen for biomarker analysis because of its non-invasive nature (Harpole et al., 2016). A great deal of evidence indicates that the urinary biomarkers reflect an individual’s metabolic and pathophysiologic state, which are also closely associated with disease activity (Aragón et al., 2018; Kowalewski and Forster, 2021). Consequently, an interesting question was raised as to the whether urinary biomarkers could be used to predict the occurrence of QTc prolongation in BDQ-treated patients. To address this concern, we conducted a prospective cohort study to assess the frequency and severity of QTc prolongation. The ultra-high performance liquid chromatography-MS/MS (UHPLC-MS/MS) was used to generate metabolic profiling for the discovery of potential predictive urine biomarkers of QTc prolongation in BDQ-treated patients.

## Methods

### Patient recruitment and sample collection

The inclusion criteria for this study were rifampicin-resistant TB (RR-TB) and multidrug-resistant TB (MDR-TB) patients who received the BDQ regimen from April 2022 to June 2023 at Beijing Chest Hospital, Capital Medical University, and had complete medical records which included patient profiles, drugs used, laboratory examinations, and ECG examinations. The inclusion criteria of patients were as follows: 1) patients with RR/MDR-TB confirmed by sputum culture and drug susceptibility tests (DST) or Xpert MTB/RIF; or 2) a pathological diagnosis of RR/MDR-TB in lung specimens. RR-TB was defined as resistance to at least RIF, including rifampicin mono-resistant tuberculosis (RMR-TB) and MDR-TB (resistance to at least RIF and INH) as definition of WHO guidelines (World Health Organization, 2019). Patients with severe heart, liver, lung or kidney dysfunction or failure, malignant tumors and serum HIV positive were excluded from the study whatever QIP group or QIU group; patients with BDQ allergy, Fridericia-corrected QT (QTc F) interval >450 ms, or significant electrocardiograph abnormalities at screening (0 weeks) were excluded from the study.

All enrolled RR-TB patients were treated with regimen containing BDQ. During the therapy, the patients were subjected to corresponding laboratory examination and ECG examination, and were divided into QTc interval prolongation (QIP) group and QTc interval un-prolongation (QIU) group according to the ECG examination results. The middle of the fasting morning urine was collected at the time of diagnosis (before treatment onset, 0 weeks) and again at various intervals during the treatment phase (weeks 2, 4, and 8). Transport to the analytical laboratory from the clinical site and further storage were maintained at −80°C. Ethical approval was granted for the collection and use of these samples by the ethics committees of Beijing Chest Hospital, Capital Medical University. Informed consent from each participant for the use of their urine for research purposes was also obtained, and all samples were anonymized immediately to maintain confidentiality.

### Untargeted metabolomic analysis of urine specimens

Metabolomics profiling was analyzed using a UPLC-ESI-Q-Orbitrap-MS system (UHPLC, Shimadzu Nexera X2 LC-30AD, Shimadzu, Japan) coupled with Q-Exactive Plus (Thermo Scientific, San Jose, USA). For liquid chromatography (LC) separation, samples were analyzed using a ACQUITY UPLC^®^ HSS T3 column (2.1 × 100 mm, 1.8 μm) (Waters, Milford, MA, USA). The electrospray ionization (ESI) with positive-mode and negative mode were applied for MS data acquisition separately. The raw MS data were processed using MS-DIAL for peak alignment, retention time correction and peak area extraction. The metabolites were identified by accuracy mass (mass tolerance <10 ppm) and MS/MS data (mass tolerance <0.02Da) which were matched with HMDB, massbank and other public databases and our self-built metabolite standard library. In the extracted-ion features, only the variables having more than 50% of the nonzero measurement values in at least one group were kept.

### Targeted metabolomic analysis of urine specimens

To ensure data quality for metabolic profiling, Quality control (QC) samples were prepared by pooling aliquots of all samples that were representative of the all samples under analysis, and used for data normalization. The LC/MS portion of the platform was based on a Shimadzu Nexera X2 LC-30AD system equipped with an Acquity UPLC HSS T3 column (1.8um 2.1 × 50 mm Column, Waters) and a triple quadruple mass spectrometer (5500 QTRAP, AB SCIEX). Metabolites were detected in electrospray negative-ionization and positive-ionization mode. MultiQuant 3.0.2 software was used to extract the original MRM data of MT1000 KIT metabolites and obtain the peak area of each metabolite.

### Metabolomic data analysis

The discriminating metabolites were obtained using a statistically significant threshold of variable influence on projection (VIP) values obtained from the orthogonal partial least-square discriminant analysis (OPLS-DA) model and two-tailed Student’s t-test (*p*-value) on the normalized raw data. The *p*-value was calculated by one-way analysis of variance (ANOVA) for multiple groups analysis. Metabolites with VIP greater than 1 and *p*-value less than 0.05 were considered to be statistically significant metabolites. The identified differential metabolites were used to perform cluster analyses with R package and GraphPad Prism (version 7.0.4) was used for statistical charts and receiver operator characteristic (ROC) curves.

To identify the perturbed biological pathways, the differential metabolite data were performed KEGG pathway analysis using KEGG database (http://kegg.jp). KEGG enrichment analyses were carried out with the Fisher’s exact test, and FDR correction for multiple testing was performed. Enriched KEGG pathways were nominally statistically significant at the *p* < 0.05 level. Quantitative variables were expressed as the means ± standard deviations (SD) or median interquartile range (IQR) values and compared using Student’s t-test. QTc F is QT interval corrected using the Fridericia formula. Statistical analyses were performed using SPSS version 26.0 (SPSS Institute, IL, USA), and statistical significance was set at *p* < 0.05.

## Results

### Study design and patients

**FIGURE 1:**
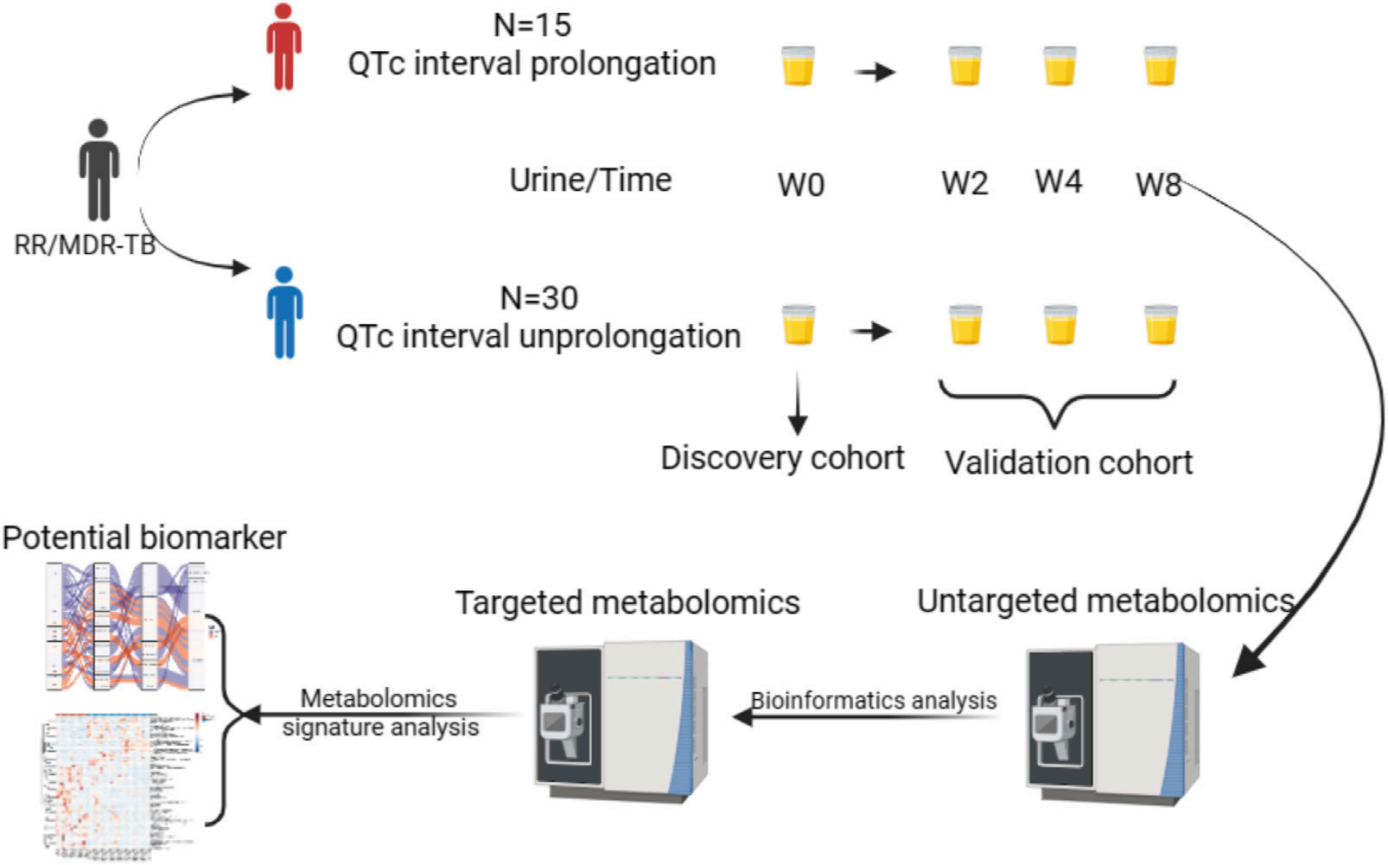
Study overview.

In this study, 15 RR/MDR-TB patients with QTc interval prolongation (QIP) and 30 RR/MDR-TB patients with QTc interval un-prolongations (QIU) were recruited when RR-TB was treated with BDQ regimen, and urine was collected at 0, 2, 4, and 8 weeks. The sampling of patient urine collected at the time of diagnosis (0 weeks) were included in discovery cohort for metabolic pathways and biomarker selection, and urine samples from treatment phases (weeks 2, 4, and 8) were included in validation cohort to verify the metabolic pathways and biomarkers. Untargeted metabolomics techniques were used to explore the metabolic pathways associated with prolonged QTc interval among the BDQ-treated patients. Furthermore, to identify potential predictive urine biomarkers for QTc prolongation in patients, we applied targeted metabolomics techniques to validate and generate metabolic profiling. The specific flow diagram and the detailed characteristics of recruited participants are shown in Figure 1 and Supplementary Table S1.

### Identification of metabolite pathway involved in QTc prolongation by untargeted metabolomics analysis

**FIGURE 2:**
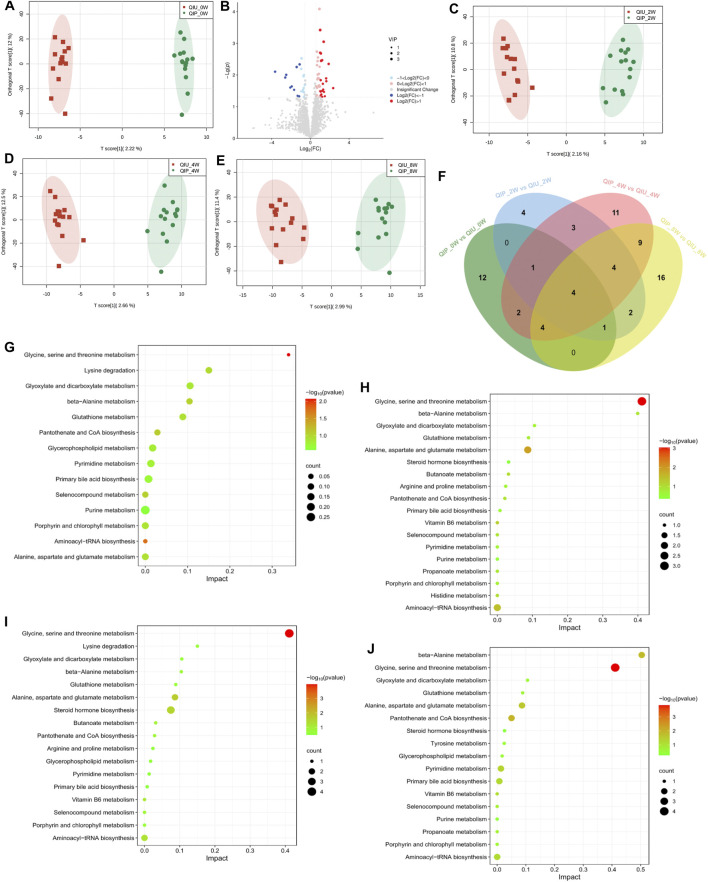
Untargeted metabolomic signatures of patients with RR-TB in BDQ-treated Scatter plot of OPLS-DA model: **(A)** QIP_0W vs QIU_0W: R^2^X:0.256, R^2^Y:0.991, Q2:0.183; **(C)** QIP_2W vs QIU_2W:R^2^X:0.331, R^2^Y:0.982, Q2:0.0549; **(D)** QIP_4W vs QIU_4W: R^2^X:0.266, R^2^Y:0.981, Q2:0.291; **(E)** QIP_8W vs QIU_8W: R^2^X:0.297, R^2^Y:0.965, Q2:0.295. KEGG analysis of the differential expression of metabolites:**(G)**: QIP_0W vs QIU_0W; **(H)**: QIP_2W vs QIU_2W; **(I)**: QIP_4W vs QIU_4W; **(J)**: QIP_8W vs QIU_8W. **(B)** Volcano map of differential metabolites classification of the QIP_0W vs QIU_0W. **(F)** Venn diagram displays the number of differentially expressed metabolites in discovery cohort compared to validation cohort. (|FC| >1.5, *p* < 0.05, VIP >1).

In this study, urine samples from the discovery cohort (0 weeks) were screened by using UHPLC -MS/MS. In the OPLS-DA analysis, the samples can be completely separated from QIUs, illustrating evident differences in their urine metabolite profiles (Figure 2A). Volcano plots from untargeted metabolomic analyses highlight the different metabolites that increased (red) or decreased (blue) in the urine of QIPs, as compared to QIUs (Figure 2B). Urine samples from the validation cohort (weeks 2, 4 and 8) were analyzed by using the OPLS-DA method. According to the characteristics of the model, the data of any two groups could be well distinguished (Figures 2C–E). The overlapping differentially expressed metabolites between QIPs and QIUs were then selected for pathogenesis analysis (Figure 2F). Interestingly, we found that most differentially expressed metabolites in QIPs were concentrated in amino acids and lipids (Figure 2F). This result indicates that QTc interval prolongation in RR-TB patients with BDQ-treated mainly affected amino acid and lipid metabolism, which might play an important role in the prolongation of QTc.

To obtain further metabolic pathway information about the different metabolites, we combined the data of discovery and validation cohort into KEGG pathway Database for signal pathway analysis and performed further enrichment and statistical analysis of these results. As shown in Figure 2G, these are 14 disturbed metabolic pathways found to be discovery cohort. The main affected pathways are Glycine, serine and threonine metabolism, Glycerophospholipid metabolism and Purine metabolism. The detailed information of pathways analysis is shown in Supplementary Table S2. After KEGG enrichment analysis, the main metabolic pathways of the validation cohort in these differential metabolites are Glycine, serine and threonine metabolism, Aminoacyl-tRNA biosynthesis, Pantothenate and CoA biosynthesis, Glycerophospholipid metabolism (Figures 2H–J; Supplementary Tables S3-S5). According to the results of bioinformatics, it can be seen that the main signal pathways are concentrated in amino acid and lipid metabolism.

### Identification and validation of urine biomarkers predictive of prolonged QTc interval in RR/MDR-TB patients

**FIGURE 3:**
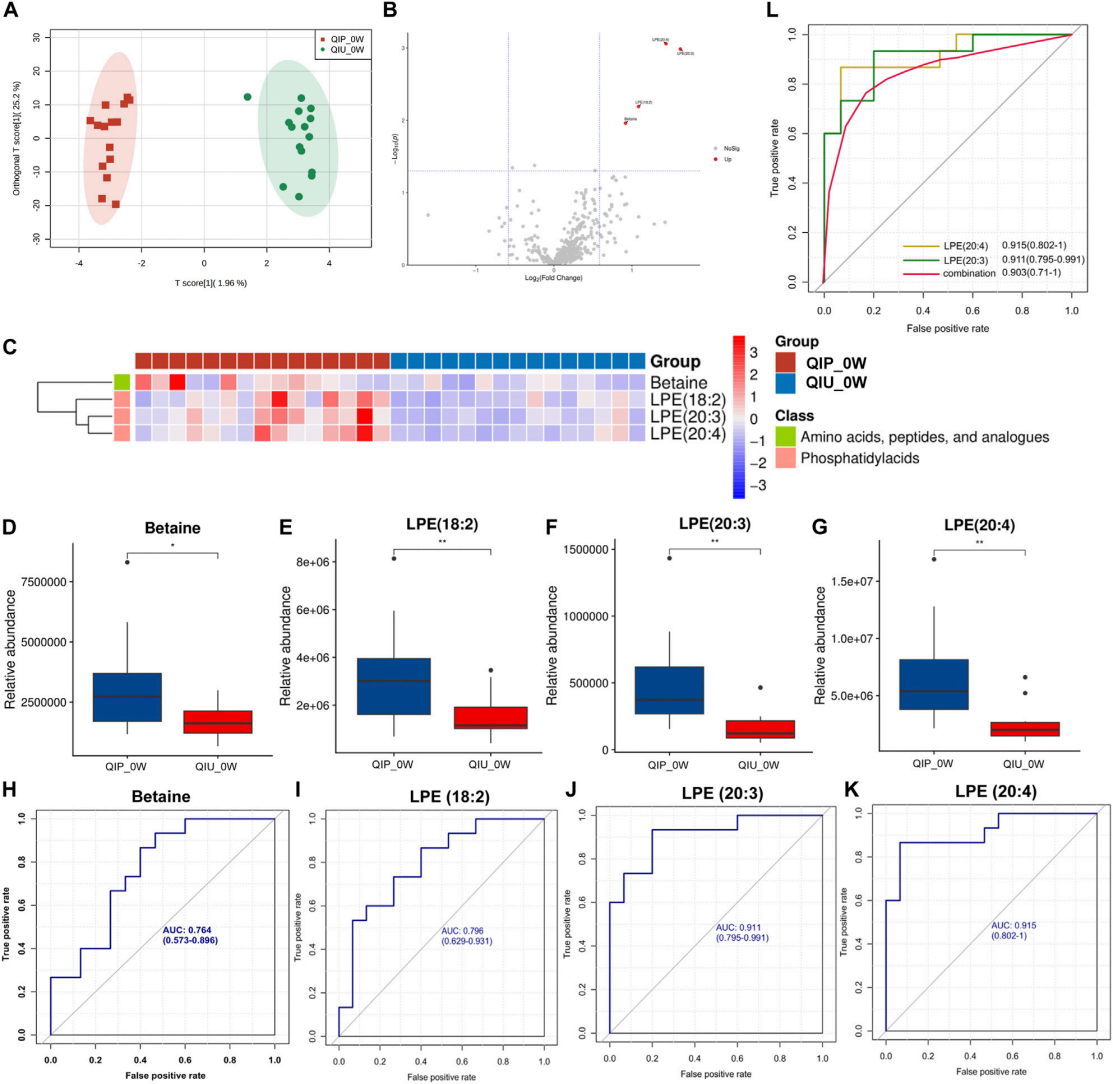
Targeted metabolomic signatures of RR-TB patients receiving BDQ-containing regimens. **(A)** OPLS-DA score plots for the QIP_0W and QIU_0W; **(B)** Volcano map for the QIP_0W and QIU_0W; **(C)** Heat map for the QIP_0W and QIU_0W; **(D–G)**: Relative abundance box plot of predicted differential metabolite biomarkers between QIP_0W and QIU_0W group; **(** **H**-**K** **)**: ROC analysis of differentially predicted metabolites between the QIP_0W and QIU_0W groups. **(L)**: Potential predictive ROC analysis of two different lipid metabolites on QTc interval prolongation: QIP_0Wvs.QIU_0W.

We investigated the possibility of differentiating QIPs from QIUs based on the molecular metabolite signatures. The quantitative data showed that the Extracted ion chromatogram had good results (Supplementary Figures S1, S2). The OPLS-DA model in the discovery cohort (0 weeks) showed that the metabolites in two groups were clearly separated (Figure 3A), which indicated that significant urine metabolites change in QIP patients. Variables with VIP value > 1.0, *p*-value < 0.05, and |FC| >1.5 were considered to be potential differential metabolites. According to the results of volcano map and heat map are shown in Figures 3B,C. After screening by a difference standard and removing exogenous metabolites, four significant differential metabolites were obtained between QIP_0W and QIU_0W, including Betaine, LPE (18:2), LPE (20:3), and LPE (20:4) (Table 1). Univariate analysis revealed significantly higher concentrations of Betaine, LPE (18:2), LPE (20:3), and LPE (20:4) observed in urine of QIPs compared with QIUs (Figures 3D–G).

**TABLE 1:** Differential metabolites between QIP_0W and QIU_0W.

Metabolite	*p*-value	FC	VIP	AUC	Sensitivity	Specificity
LPE (20:4)	0.000873	2.707232	4.056938	0.915 (0.802–1.000)	0.881 (0.827–0.936)	0.867 (0.809–0.924)
LPE (20:3)	0.001037	3.076843	4.077989	0.911 (0.795–0.991)	0.785 (0.716–0.854)	0.844 (0.783–0.906)
LPE (18:2)	0.006451	2.120300	3.508584	0.796 (0.629–0.931)	0.704 (0.627–0.781)	0.696 (0.619–0.774)
Betaine	0.010951	1.888578	3.407322	0.764 (0.573–0.896)	0.593 (0.510–0.675)	0.844 (0.783–0.906)

We performed ROC curve analysis, 95% CI value calculation, sensitivity and specificity analyses on the differential metabolites in the discovery cohort according to the aforementioned results to evaluate the prediction effect of each differential metabolite. Among them, the AUC value of Betaine was 0.764 (0.573–0.896) with 59.3% sensitivity and 84.4% specificity (Figure 3H; Table 1); the AUC value of LPE (18:2) was 0.796 (0.629–0.931) with 70.4% sensitivity and 69.6% specificity (Figure 3I; Table 1); the AUC value of LPE (20:3) was 0.911 (0.795–0.991) with 78.5% sensitivity and 84.4% specificity (Figure 3J; Table 1); and the AUC value of LPE (20:4) was 0.915 (0.802–1.000) with 88.1% sensitivity and 86.7% specificity (Figure 3K; Table 1). Based on clinical practicability and feasibility, for the predictive effect evaluation of each differential metabolite, we focus on the differential metabolites that was specifically increased in QTc interval prolongation group after BDQ exposure. On the other hand, according to the comprehensive consideration of the AUC value, 95% CI, sensitivity, and specificity of each different metabolite, LPE (20:3) and LPE (20:4) were finally selected for further predictive effect evaluation and analysis. In order to evaluate the predictive capacity of the two potential markers in combination, we used the logistic regression method to obtain an integrated prediction model with a sensitivity of 87.4%, a specificity of 78.5%, and the AUC value of 0.903 (95% CI 0.710–1.000) (Figure 3L; Table 2).

**TABLE 2:** Potential predictive ROC analysis of two different lipid metabolites on QTc interval prolongation.

(W)	Metabolite	*p*-value	FC	VIP	AUC	Sensitivity	Specificity
0	LPE (20:4)	0.000873	2.707232	4.056938	0.915 (0.802–1.000)	0.881 (0.827–0.936)	0.867 (0.809–0.924)
	LPE (20:3)	0.001037	3.076843	4.077989	0.911 (0.795–0.991)	0.785 (0.716–0.854)	0.844 (0.783–0.906)
	LPE (20:3) plus LPE (20:4)				0.903 (0.710–1.000)	0.874 (0.818–0.930)	0.785 (0.716–0.854)
2	LPE (20:4)	7.07973E-05	1.999535599	2.818594745	1.000 (1.000–1.000)	1.000 (1.000–1.000)	1.000 (1.000–1.000)
	LPE (20:3)	0.035714899	3.022346765	1.852068323	0.850 (0.600–1.000)	1.000 (1.000–1.000)	0.800 (0.598–1.000)
	LPE (20:3) plus LPE (20:4)				0.944 (0.290–1.000)	1.000 (1.000–1.000)	1.000 (1.000–1.000)
4	LPE (20:4)	9.78585E-06	2.760230383	2.613114904	0.978 (0.911–1.000)	0.881 (0.827–0.936)	0.844 (0.739–0.950)
	LPE (20:3)	0.000113452	3.108712573	2.524455978	0.944 (0.822–1.000)	0.874 (0.818–0.930)	1.000 (1.000–1.000)
	LPE (20:3) plus LPE (20:4)				0.941 (0.864–1.000)	0.859 (0.801–0.918)	1.000 (1.000–1.000)
8	LPE (20:4)	0.001930543	2.182727373	3.281488946	0.830 (0.633–0.976)	0.636 (0.352–0.921)	1.000 (1.000–1.000)
	LPE (20:3)	0.041070619	2.216148021	2.059779512	0.733 (0.515–0.912)	0.909 (0.739–1.000)	0.667 (0.428–0.905)
	LPE (20:3) plus LPE (20:4)				0.748 (0.421–1.000)	0.636 (0.352–0.921)	1.000 (1.000–1.000)

To understand the biological significance of differential metabolites in QTc interval prolonging and validate the untargeted metabolic outcomes and selected predicted metabolites. Urine samples from validation cohort (weeks 2, 4, and 8) were analyzed using targeted metabolomics techniques. In OPLS-DA score plots, it is found that there are significant biochemical differences between QIP and QIU group in the discovery and validation cohort respectively (Figure 3A; Supplementary Figure S3). The heat maps and volcano maps are shown in Supplementary Figures S4, S5, after screening by a difference standard, 19, 43, and 10 significantly differential metabolites were obtained among QIP_2W vs QIU_2W, QIP_4W vs QIU_4W, and QIP_8W vs QIU_8W, respectively (Supplementary Tables S6–S8). For the differential metabolites screened out in the analysis, we conducted a bioinformatics analysis on the differential metabolites. We imported these metabolites one by one into the KEGG database for signal pathway analysis and performed further enrichment and statistical analysis of these results. The differential abundance and the clustering analysis of differential metabolites in discovery and validation cohort were visualized in the heatmap. After KEGG enrichment analysis, the main metabolic pathways in these differential metabolites are amino acids, peptides, and analogues metabolism, phosphatidylacids metabolism (Figure 3C; Supplementary Figure S4). According to the results of bioinformatics, it can be seen that the main signal pathways are concentrated in lipid metabolism, which is in good agreement with the untargeted metabolomics results.

**FIGURE 4:**
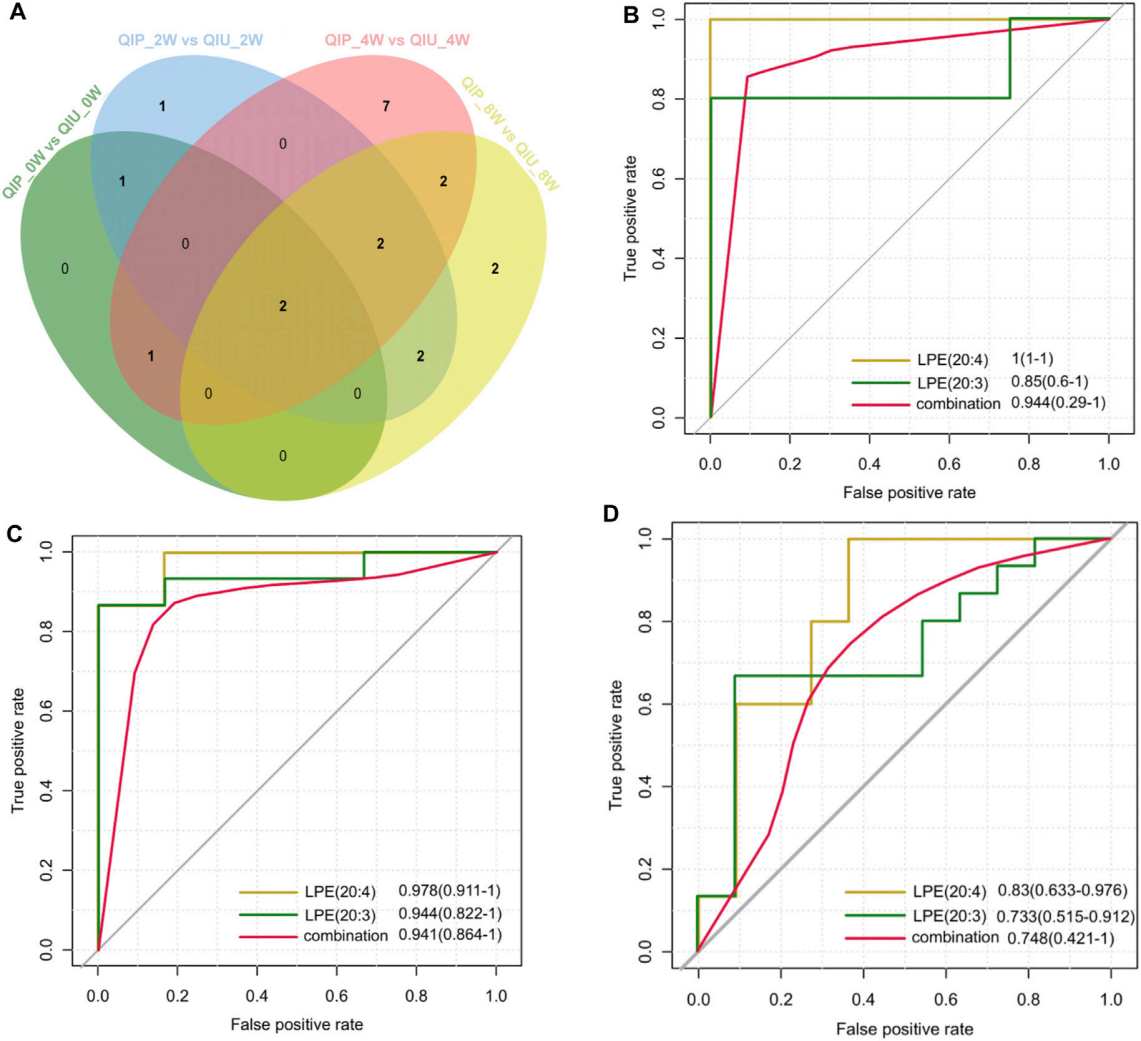
Metabolite validation for predicting QTc interval prolongation in BDQ-treated patients. **(A)** Venn diagram displays the number of differentially predicted metabolites in discovery cohort compared to validation cohort. (|FC| >1.5, *p* < 0.05, VIP >1). Potential predictive ROC analysis of two different lipid metabolites on QTc interval prolongation:**(B)**: QIP_2Wvs.QIU_2W; **(C)**: QIP_4W vs QIU_4W; **(D)**: QIP_8W vs QIU_8W.

To evaluate the reliability of predictive biomarkers, data from urine samples in the discovery and validation cohort were analyzed. Figure 4A shows the Venn diagrams that highlight the overlapping and unique metabolites predicting QTc interval prolongation in BDQ-treated across the different groups. Of these, 2 were overlapping for LPE (20:3) and LPE (20:4). At 2 weeks, the AUC value of LPE (20:3) was 0.850 (0.600–1.000) with 100% sensitivity and 80% specificity; the AUC value of LPE (20:4) was 1 (1.000–1.000) with 100% sensitivity and 100% specificity; the AUC value of LPE (20:3) plus LPE (20:4) was 0.944 (0.290–1.000) with 100% sensitivity and 100% specificity (Table 2; Figure 4B). At 4 weeks, the AUC value of LPE (20:3) was 0.944 (0.822–1.000) with 87.4% sensitivity and 100% specificity; the AUC value of LPE (20:4) was 0.978 (0.91–1.000) with 88.1% sensitivity and 84.4% specificity; the AUC value of LPE (20:3) plus LPE (20:4) was 0.941 (0.864–1.000) with 85.9% sensitivity and 100% specificity (Table 2; Figure 4C). At 8 weeks, the AUC value of LPE (20:3) was 0.733 (0.515–0.912) with 90.9% sensitivity and 66.7% specificity; the AUC value of LPE (20:4) was 0.830 (0.633–0.976) with 63.6% sensitivity and 100% specificity; the AUC value of LPE (20:3) plus LPE (20:4) was 0.748 (0.421–1.000) with 63.6% sensitivity and 100% specificity (Table 2; Figure 4D).

## Discussion

The major adverse event related to BDQ administration is cardiac QTc interval prolongation. The incidence of QTc interval prolongation is associated with an elevated level of BDQ’s main metabolite (M2) (Food and Drug Administration, 2012; Janssen Therapeutics, 2012). Besides, the QTc interval is subjected to intrinsic variability across multiple individual factors, such as sex, age, body mass index or food intake (Piotrovsky, 2005). These findings emphasize the important role of inter-individual variation of metabolism in the incidence of QTc interval prolongation. In this study, we compared the metabolic profiling of BDQ-treated patients with or without QTc interval prolongation by a non-targeted metabolomic analysis. We firstly identified several biomarkers that can predict the occurrence of this adverse event for RR/MDR-TB patients, including Betaine, LPE (18:2), LPE (20:3) and LPE (20:4). Notably, the combined use of LPE (20:3) and LPE (20:4) yielded a sensitivity of 87.4% and a specificity of 78.5%. With the progression of BDQ treatment, the abundance of these two LPEs significantly increased among patients experiencing QTc interval prolongation *versus* cardiac event-naïve patients, indicating the promising performance of these biomarkers for predicting and monitoring the QTc interval prolongation among BDQ-treated patients.

Lysophospholipids play a pivotal role in the maintenance of cell membrane stability and signal transduction in host cells (Chorell et al., 2020). Interestingly, LPEs have been shown to be altered in patients with QTc interval prolongation. Despite being signaling molecules, they could be toxic to cells, as well as myocardial cells, at high concentration, via disrupting membrane structure and causing cell lysis (Tan et al., 2020). In addition, LPE was shown to involve in calcium influx in a G-protein-coupled receptor-dependent manner (Lee et al., 2017). Considering the importance of calcium regulation in myocardial repolarization (Arking et al., 2014), the LPE-induced increase of intracellular calcium may be another plausible explanation for the correlation between LPE and QTc interval prolongation in TB patients. Taken together, the elevated level of LPEs may reflect high susceptibility to heart diseases. In line with our hypothesis, an experimental study by Duflot and colleagues found that circulating LPE species were increased in rats with cardiovascular disease, suggesting their role in the pathophysiology of this disease (Duflot et al., 2021). Further studies are warranted to elucidate the underlying molecular mechanisms of LPEs in predicting cardiovascular adverse events.

Another interesting finding of our study was that only LPEs, especially polyunsaturated LPEs, were identified as predictive biomarkers associated with QTc interval prolongation, whereas no significant differences were identified in other bioactive phospholipids between two groups. The predominance of LPE emphasizes the interruption of *in vivo* phospholipid homeostasis, specifically the ratio between ethanolamine- and choline-containing phospholipids, thereby in turn negatively impacting vascular wall function, tissue lipid mobilization and calcium homeostasis (Meikle and Summers, 2017). The *in vivo* production of LPE mainly depends on the hydrolysis of phosphatidylethanolamine by phospholipase A2 (PLA2) in the inner leaflet of plasma membrane. This raises interesting questions as to the cell types and tissues that produce circulating LPE. Interestingly, the PLA2 family also play multiple functions in anti-bacterial infection and inflammation (Hurley and McCormick, 2008). A previous study has demonstrated that the MTB-infected phagocytes could facilitate the clearance of intracellular tubercle bacilli via induction of PLA2-meditated apoptosis (Duan et al., 2001). Although the exact mechanism remains unclear, we speculate that the phagocytes may serve as an intracellular pool of LPEs in individuals infected with MTB. Further experiments are required to validate our hypothesis.

In addition, the LPEs exhibited persistent differences in the BDQ-treated TB patients experiencing QTc interval prolongation. This phenomenon was associated with the incidence of cardiovascular events. Notably, a prospective nested case-control study from Sweden found the simultaneous coexistence of the elevated LPE levels with increased proinflammation cytokines in the plasma samples of non-ST-elevation myocardial infarction patients (Chorell et al., 2020), indicating the potential correlation between proinflammation cytokines and lysophospholipid metabolism. Recently, besides strong bactericidal effect against tubercle bacilli, BDQ potentially exerts immunomodulatory effects by inducing high-level proinflammation cytokine expression (Lyu et al., 2022). Therefore the immunomodulatory role of BDQ in host macrophages may be another underlying mechanism that remodels lipid metabolism and triggers the emergence of QTc interval prolongation.

We also acknowledged several limitations to the present study. First, despite enrolment of all patients meeting the inclusion criteria in the study, the small sample size of RR/MDR-TB patients with QTc interval prolongation weakened the significance of our conclusion. Second, besides BDQ, several other anti-TB drugs were also associated with QTc interval prolongation, such as clofazimine and moxifloxacin (Dooley et al., 2021). It was uncertain whether the polyunsaturated LPEs could be used to predict the cardiovascular adverse events in these individuals. Third, there was a strong correlation in metabolite levels between plasma and urine (Bi et al., 2022). However, due to the difficulty to obtain blood samples, we did not compare the lipid metabolic state in circulating blood. Nevertheless, our results firstly investigated the predictive biomarkers in urine via mass spectrometry-based non-targeted metabolic profiling, which will bring new insights in early identification of TB patients at high risk of developing QTc interval prolongation.

To conclude, our data demonstrate that the combined use of LPE (20:3) and LPE (20:4) yielded a sensitivity of 87.4% and a specificity of 78.5% for predicting the occurrence of QTc interval prolongation in BDQ-treated patients. With the progression of BDQ treatment, the abundance of these two LPEs significantly increased among patients experiencing QTc interval prolongation *versus* cardiac event-naïve patients. Further studies are warranted to investigate the underlying mechanism in the relationship between the elevated LPEs and QTc interval prolongation.

## Data Availability

The datasets presented in this study can be found in online repositories. The names of the repository/repositories and accession number(s) can be found in the article/Supplementary Material.
