# Supplementary material for: Identification of urine biomarkers predictive of prolonged QTc interval in multidrug-resistant tuberculosis patients treated with bedaquiline
Source: Front Pharmacol. 2024 May 30;15:1362544. doi: 10.3389/fphar.2024.1362544 (PMC11169739; doi:10.3389/fphar.2024.1362544)
Supplement: Supplementary file 1 [file DataSheet1.docx]

| Table S1. Statistics of clinical characteristics and laboratory indexes of the study cohort | | |
| --- | --- | --- |
|  | QTc interval prolongation(N=15) | No QTc interval prolongation(N=30) |
| Sex, no. (%) | | |
| male | 8 (53.3%) | 21(70.0%) |
| female | 7(46.7%) | 9(30.0%) |
| Age, year | | |
| Mean ± SD | 35.1±11.3 | 38.6±14.8 |
| Median (IQR) | 35.0（25.0-39.0） | 35.0（25.8-54.5） |
| Range | 21.0-58.0 | 19.0-65.0 |
| BMI, kg/m^2^ | | |
| Mean ± SD | 20.6±2.2 | 20.4±3.1 |
| Median (IQR) | 20.3（19.1-22.7） | 20.4（18.6-22.3） |
| Range | 16.7-23.7 | 13.6-27.7 |
| Symptoms, no. (%) | | |
| Cough | 12(80%) | 26(86.67%) |
| Expectoration | 12(80%) | 26(86.67%) |
| Hemoptysis | 2(13.33%) | 6(20%) |
| Chest pain | 9(60%) | 8(26.67%) |
| Dyspnea | 3(20%) | 3(10%) |
| Fatigue | 4(26.67%) | 7(23.33%) |
| Weight loss | 2(13.33%) | 2(6.67%) |
| fever | 4(26.67%) | 10(33.33%) |
| Comorbidity, no. (%) | 9(60%) | 26(86.67%) |
| Sputum Acid Fast Bacilli Smear, no. (%) | | |
| Positive | 5(33.33%) | 13(43.33%) |
| Negative | 10(66.67%) | 17(56.67%) |
| Indeterminate | - | - |
| Sputum Culture, no. (%) | | |
| Positive | 14(93.33%) | 26(86.67%) |
| Negative | 1(6.67%) | 4(13.33%) |
| Indeterminate | - | - |
| GeneXpert/MTB RIF assay, no. (%) | | |
| Positive | 15(100%) | 29(96.67%) |
| Negative | 0(0%) | 1(3.33%) |
| Indeterminate | - | - |
| Drug resistance, no. (%) | | |
| RR-TB | 7(46.67%) | 3(10%) |
| MDR | 6(40%) | 22(73.33%) |
| Pre-XDR-TB | 2(13.33%) | 5(16.67%) |
| Baseline QTc (ms) | | |
| Mean ± SD | 404.2±16.2 | 398.3±22.6 |
| Median (IQR) | 404.0（389.0-419.0） | 402.0（384.5-412.3） |
| Range | 377.0-427.0 | 332.0-433.0 |
| Background regimen, no. (%) | | |
| Moxifloxacin | 8.0(53.3%) | 12.0(40.0%) |
| Levofloxacin | 1.0(6.7%) | 6.0(20.0%) |
| Linezolid | 13.0(86.7%) | 27.0(90.0%) |
| Contezolid | 2.0(13.3%) | 1.0(3.3%) |
| Clofazimine | 15.0(100.0%) | 29.0(96.7%) |
| Cycloserine | 14.0(93.3%) | 27.0(90.0%) |
| Amikacin | 2.0(13.3%) | 4.0(13.3%) |

IQR: interquartile range; SD: standard deviation; BMI: Body Mass Index；RR-TB: rifampicin-resistant tuberculosis；MDR-TB: multidrug-resistant tuberculosis; Pre-XDR: pre-extensively drug-resistant tuberculosis.

Table S2. Disturbed metabolic pathways in QIP_0 week vs. QIU_0 week

|  | Total | Hits | Raw p | Impact | FDR |
| --- | --- | --- | --- | --- | --- |
| Glycine, serine and threonine metabolism | 33 | 2 | 0.008638 | 0.3387 | 0.72555 |
| Aminoacyl-tRNA biosynthesis | 48 | 2 | 0.017861 | 0 | 0.75016 |
| Pantothenate and CoA biosynthesis | 19 | 1 | 0.082869 | 0.02857 | 1 |
| Selenocompound metabolism | 20 | 1 | 0.087063 | 0 | 1 |
| beta-Alanine metabolism | 21 | 1 | 0.091239 | 0.10448 | 1 |
| Lysine degradation | 25 | 1 | 0.10778 | 0.15023 | 1 |
| Alanine, aspartate and glutamate metabolism | 28 | 1 | 0.12002 | 0 | 1 |
| Glutathione metabolism | 28 | 1 | 0.12002 | 0.08873 | 1 |
| Porphyrin and chlorophyll metabolism | 30 | 1 | 0.1281 | 0 | 1 |
| Glyoxylate and dicarboxylate metabolism | 32 | 1 | 0.13611 | 0.10582 | 1 |
| Glycerophospholipid metabolism | 36 | 1 | 0.15195 | 0.01736 | 1 |
| Pyrimidine metabolism | 39 | 1 | 0.16367 | 0.01318 | 1 |
| Primary bile acid biosynthesis | 46 | 1 | 0.19047 | 0.00758 | 1 |
| Purine metabolism | 65 | 1 | 0.25953 | 0 | 1 |

Table S3. Disturbed metabolic pathways in QIP_2 week vs. QIU_2 week

|  | Total | Hits | Raw p | Impact | FDR |
| --- | --- | --- | --- | --- | --- |
| Glycine, serine and threonine metabolism | 33 | 3 | 0.000954 | 0.4113 | 0.080141 |
| Alanine, aspartate and glutamate metabolism | 28 | 2 | 0.012953 | 0.08654 | 0.54401 |
| Aminoacyl-tRNA biosynthesis | 48 | 2 | 0.036071 | 0 | 1 |
| Vitamin B6 metabolism | 9 | 1 | 0.056731 | 0 | 1 |
| Butanoate metabolism | 15 | 1 | 0.092925 | 0.03175 | 1 |
| Histidine metabolism | 16 | 1 | 0.098834 | 0 | 1 |
| Pantothenate and CoA biosynthesis | 19 | 1 | 0.11636 | 0.02143 | 1 |
| Selenocompound metabolism | 20 | 1 | 0.12213 | 0 | 1 |
| beta-Alanine metabolism | 21 | 1 | 0.12786 | 0.39925 | 1 |
| Propanoate metabolism | 23 | 1 | 0.13924 | 0 | 1 |
| Glutathione metabolism | 28 | 1 | 0.16709 | 0.08873 | 1 |
| Porphyrin and chlorophyll metabolism | 30 | 1 | 0.17801 | 0 | 1 |
| Glyoxylate and dicarboxylate metabolism | 32 | 1 | 0.18879 | 0.10582 | 1 |
| Arginine and proline metabolism | 38 | 1 | 0.22038 | 0.02385 | 1 |
| Pyrimidine metabolism | 39 | 1 | 0.22554 | 0 | 1 |
| Primary bile acid biosynthesis | 46 | 1 | 0.26078 | 0.00758 | 1 |
| Purine metabolism | 65 | 1 | 0.34928 | 0 | 1 |
| Steroid hormone biosynthesis | 85 | 1 | 0.43203 | 0.03203 | 1 |

Table S4. Disturbed metabolic pathways in QIP_4 week vs. QIU_4 week

|  | Total | Hits | Raw p | Impact | FDR |
| --- | --- | --- | --- | --- | --- |
| Glycine, serine and threonine metabolism | 33 | 4 | 0.000107 | 0.4113 | 0.008954 |
| Alanine, aspartate and glutamate metabolism | 28 | 2 | 0.021712 | 0.08654 | 0.85479 |
| Steroid hormone biosynthesis | 85 | 3 | 0.030528 | 0.07475 | 0.85479 |
| Aminoacyl-tRNA biosynthesis | 48 | 2 | 0.058948 | 0 | 1 |
| Vitamin B6 metabolism | 9 | 1 | 0.073183 | 0 | 1 |
| Butanoate metabolism | 15 | 1 | 0.11919 | 0.03175 | 1 |
| Pantothenate and CoA biosynthesis | 19 | 1 | 0.14868 | 0.02857 | 1 |
| Selenocompound metabolism | 20 | 1 | 0.15591 | 0 | 1 |
| beta-Alanine metabolism | 21 | 1 | 0.16308 | 0.10448 | 1 |
| Lysine degradation | 25 | 1 | 0.19121 | 0.15023 | 1 |
| Glutathione metabolism | 28 | 1 | 0.21173 | 0.08873 | 1 |
| Porphyrin and chlorophyll metabolism | 30 | 1 | 0.22514 | 0 | 1 |
| Glyoxylate and dicarboxylate metabolism | 32 | 1 | 0.23835 | 0.10582 | 1 |
| Glycerophospholipid metabolism | 36 | 1 | 0.26413 | 0.01736 | 1 |
| Arginine and proline metabolism | 38 | 1 | 0.27672 | 0.02385 | 1 |
| Pyrimidine metabolism | 39 | 1 | 0.28293 | 0.01318 | 1 |
| Primary bile acid biosynthesis | 46 | 1 | 0.3251 | 0.00758 | 1 |

Table S5. Disturbed metabolic pathways in QIP_8 week vs. QIU_8 week

|  | Total | Hits | Raw p | Impact | FDR |
| --- | --- | --- | --- | --- | --- |
| Glycine, serine and threonine metabolism | 33 | 4 | 0.000147 | 0.4113 | 0.012348 |
| Pantothenate and CoA biosynthesis | 19 | 2 | 0.011871 | 0.05 | 0.404 |
| beta-Alanine metabolism | 21 | 2 | 0.014428 | 0.50373 | 0.404 |
| Alanine, aspartate and glutamate metabolism | 28 | 2 | 0.02505 | 0.08654 | 0.52605 |
| Pyrimidine metabolism | 39 | 2 | 0.046401 | 0.01318 | 0.77954 |
| Primary bile acid biosynthesis | 46 | 2 | 0.062521 | 0.00758 | 0.80931 |
| Aminoacyl-tRNA biosynthesis | 48 | 2 | 0.067442 | 0 | 0.80931 |
| Vitamin B6 metabolism | 9 | 1 | 0.07861 | 0 | 0.82541 |
| Selenocompound metabolism | 20 | 1 | 0.16689 | 0 | 1 |
| Propanoate metabolism | 23 | 1 | 0.18957 | 0 | 1 |
| Glutathione metabolism | 28 | 1 | 0.22609 | 0.08873 | 1 |
| Porphyrin and chlorophyll metabolism | 30 | 1 | 0.24027 | 0 | 1 |
| Glyoxylate and dicarboxylate metabolism | 32 | 1 | 0.2542 | 0.10582 | 1 |
| Glycerophospholipid metabolism | 36 | 1 | 0.28136 | 0.01736 | 1 |
| Tyrosine metabolism | 42 | 1 | 0.32039 | 0.02463 | 1 |
| Purine metabolism | 65 | 1 | 0.45248 | 0 | 1 |
| Steroid hormone biosynthesis | 85 | 1 | 0.54752 | 0.02562 | 1 |

Table S6. Differential metabolites between QIP_2W and QIU_2W

| **Metabolite** | **P value** | **FC** | **VIP** | **AUC** |
| --- | --- | --- | --- | --- |
| LPE(20:4) | 7.07973E-05 | 1.999535599 | 2.818594745 | 1(1-1) |
| CEA | 0.004771675 | 1.953393586 | 2.699048237 | 0.95(0.833-1) |
| PE(18:1/22:5) | 0.006265289 | 2.629444709 | 2.455900561 | 0.883(0.708-1) |
| N-Carbamoyl-L-aspartate | 0.028425791 | 1.950750788 | 2.472318468 | 0.883(0.667-1) |
| Choline | 0.0474371 | 1.535049822 | 2.100089019 | 0.817（0.55-1） |
| TMP | 0.006448063 | 2.001014177 | 2.472892525 | 0.867（0.6-1） |
| 3-Hydroxyhexanoylcarnitine | 0.009495813 | 1.953813377 | 2.379355365 | 0.867（0.667-1） |
| 5-Hydroxyindole-3-acetic acid | 0.02263702 | 1.871211084 | 2.315545025 | 0.867（0.7-1） |
| D-Fructose 6-phosphate | 0.02990498 | 1.828272627 | 2.01083497 | 0.85（0.6-1） |
| LPE(20:3) | 0.035714899 | 3.022346765 | 1.852068323 | 0.85（0.6-1） |
| N6-glycerinyl | 0.016739255 | 1.954850757 | 2.299316163 | 0.817（0.491-1） |
| Dehydrolithocholic acid | 0.016915578 | 2.381775712 | 2.162957235 | 0.817（0.567-0.983） |
| PG(18:0/20:2) | 0.032900824 | 2.03522834 | 2.085770079 | 0.817（0.575-0.983） |
| Enterolactone | 0.043899942 | 2.024063514 | 1.974115409 | 0.8（0.533-1） |
| Oxoglutaric acid | 0.010251075 | 5.367274593 | 1.98086025 | 0.783（0.483-1） |
| Inositol | 0.040970125 | 1.65453312 | 2.003669401 | 0.783（0.517-1） |
| Pimelylcarnitine | 0.03241414 | 2.60068952 | 2.112302453 | 0.733（0.3-1） |
| 2-Ketoglutaric acid | 0.009584032 | 5.9119474 | 1.990187259 | 0.7（0.308-1） |
| Hexose Disaccharide Pool | 0.023010638 | 3.39496877 | 2.110229687 | 0.7（0.3-1） |

Table S7. Differential metabolites between QIP_4W and QIU_4W

| **Metabolite** | **P value** | **FC** | **VIP** | **AUC** |
| --- | --- | --- | --- | --- |
| LPE(20:4) | 9.78585E-06 | 2.760230383 | 2.613114904 | 0.978(0.911-1) |
| LPE(20:3) | 0.000113452 | 3.108712573 | 2.524455978 | 0.944(0.822-1) |
| Trimethylamine N-oxide | 0.000559627 | 2.775685122 | 2.517808371 | 0.922(0.789-1) |
| Gamma-Aminobutyric acid | 0.000640327 | 2.483046687 | 2.568881143 | 0.922(0.794-1) |
| Glycine | 0.000960537 | 2.25309491 | 2.450314064 | 0.9(0.733-1) |
| Choline | 0.003756165 | 1.866372408 | 2.220229778 | 0.822(0.511-1) |
| Serotonin | 0.004666872 | 1.767789076 | 2.078148314 | 0.811(0.556-0.967) |
| Cyclic adenosine monophosphate | 0.005603496 | 1.563661942 | 2.280205763 | 0.878(0.7-1) |
| Deoxyguanosine | 0.005769164 | 1.947371331 | 2.013601406 | 0.856(0.622-1) |
| Dimethylglycine | 0.006629111 | 1.82641 | 2.050402856 | 0.811(0.505-1) |
| PE(18:2/22:6) | 0.007596514 | 1.875018887 | 1.870446031 | 0.844(0.639-0.978) |
| NG,NG-Dimethyl-L-arginine | 0.007746559 | 1.790056158 | 1.81151626 | 0.822(0.528-1) |
| 2-Hydroxyadenine | 0.008382152 | 1.671920987 | 1.894380247 | 0.856(0.639-1) |
| LPE(18:2) | 0.00887046 | 1.750270002 | 2.040306462 | 0.856(0.667-1) |
| GALA | 0.011460815 | 2.21624113 | 1.781413642 | 0.822(0.6-0.989) |
| Quinolin-2,8-diol | 0.011517389 | 1.817540953 | 2.198828783 | 0.8(0.528-1) |
| 2-Piperidone | 0.013163143 | 2.251786462 | 2.028560846 | 0.811(0.628-0.978) |
| Purine | 0.014145273 | 1.947498732 | 1.754965178 | 0.8(0.511-1) |
| Urocanic acid | 0.014876353 | 2.339170862 | 1.965080412 | 0.8(0.488-1) |
| Tyramine | 0.01510776 | 2.332922792 | 2.015661207 | 0.789(0.511-0.978) |
| L-Carnosine | 0.017485562 | 1.817334356 | 1.651145012 | 0.8(0.556-1) |
| Dimethyl-L-arginine | 0.021065361 | 1.656796655 | 1.791028156 | 0.722(0.428-0.944) |
| S-Methyl-L-cysteine | 0.022465137 | 1.61517969 | 1.831652943 | 0.811(0.578-1) |
| 2-Ketobutyric acid | 0.02514541 | 2.513490094 | 1.727815171 | 0.811(0.616-0.967) |
| Carnitine-C14 | 0.025297784 | 2.137116144 | 1.641166333 | 0.744(0.444-0.978) |
| Mevalonic acid | 0.026106965 | 1.598948481 | 1.363706529 | 0.789(0.556-0.978) |
| D-Ala-D-ala | 0.027201677 | 1.528422619 | 2.012880744 | 0.8(0.572-0.967) |
| Cytidine | 0.027577966 | 4.513824688 | 1.494391729 | 0.711(0.394-0.939) |
| Hypoxanthine | 0.029943573 | 1.919442484 | 1.738531134 | 0.822(0.583-0.967) |
| Hordenine | 0.030698956 | 1.891492445 | 1.392513395 | 0.822(0.616-0.978) |
| Gluconolactone | 0.032236718 | 1.801833942 | 1.736738056 | 0.778(0.522-0.967) |
| Carnitine-C6DC | 0.033038391 | 1.639579861 | 1.68517265 | 0.788(0.561-0.972) |
| Nicotinamide ribotide | 0.034893191 | 1.981903653 | 1.618380795 | 0.744(0.45-0.967) |
| 5-Hydroxy-L-tryptophan | 0.036512252 | 1.778468098 | 1.612893246 | 0.744(0.467-0.933) |
| Hypotaurine | 0.036756952 | 2.394355786 | 1.756587878 | 0.822(0.622-0.967) |
| N1-Methyl-2-pyridone-5-carboxamide | 0.038272876 | 1.667748552 | 1.778564616 | 0.811(0.5-1) |
| Cer d18:1/26:0 | 0.040021989 | 1.618117006 | 1.370412535 | 0.789(0.567-0.956) |
| N-Acetylcadaverine | 0.042199336 | 2.155385461 | 1.789861597 | 0.755(0.5-0.978) |
| Carnitine-C5DC | 0.04343626 | 1.546441457 | 1.69998329 | 0.733(0.444-0.944) |
| Picolinic acid | 0.043854009 | 1.862451892 | 1.432734617 | 0.767(0.516-0.967) |
| Diphenylamine | 0.045897398 | 0.647130287 | 1.429548416 | 0.767(0.489-1) |
| D-Glucuronic acid | 0.048698732 | 1.898866438 | 1.538841587 | 0.811(0.611-0.956) |
| Glycyl-L-leucine | 0.049542668 | 1.548395283 | 1.371031894 | 0.756(0.5-0.956) |

Table S8. Differential metabolites between QIP_8W and QIU_8W

| **Metabolite** | **P value** | **FC** | **VIP** | **AUC** |
| --- | --- | --- | --- | --- |
| L-Homoserine | 0.002409183 | 1.986151306 | 3.375843305 | 0.842（0.657-1） |
| LPE(20:4) | 0.001930543 | 2.182727373 | 3.281488946 | 0.83（0.633-0.976） |
| Threonine | 0.004530729 | 1.830466147 | 3.126192978 | 0.812（0.621-0.964） |
| Eicosapentaenoic acid | 0.032107435 | 0.573211372 | 2.817268403 | 0.745（0.56-0.927） |
| 3,7,12 Taurodehydrocholic acid | 0.019615206 | 0.655287464 | 2.568629407 | 0.77（0.56-0.955） |
| 2-Piperidone | 0.030994923 | 1.556635595 | 2.376245443 | 0.764（0.545-0.921） |
| Bilirubin | 0.038775867 | 0.441768967 | 2.312508301 | 0.806（0.655-0.93） |
| Cholestenone | 0.030908938 | 0.576354732 | 2.143806393 | 0.73（0.488-0.888） |
| LPE(20:3) | 0.041070619 | 2.216148021 | 2.059779512 | 0.733（0.515-0.912） |
| 5'-Methylthioadenosine | 0.0425012 | 0.607119806 | 1.848790036 | 0.824（0.615-0.964） |


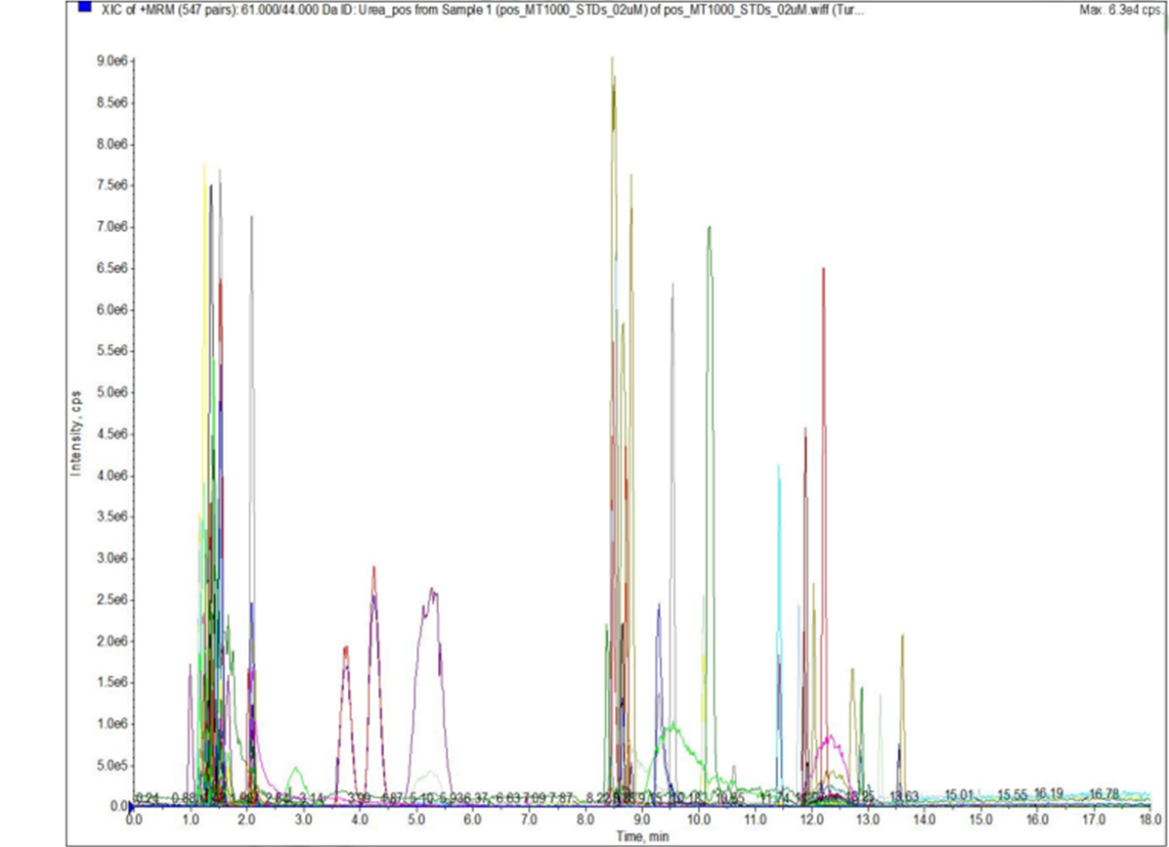


Figure S1. Extracted ion chromatogram (XIC) of mixed samples in positive ion mode.

The abscissa is the retention time (RT) of metabolites detection, and the ordinate is the ion current intensity of the detection (CPS, count per second).


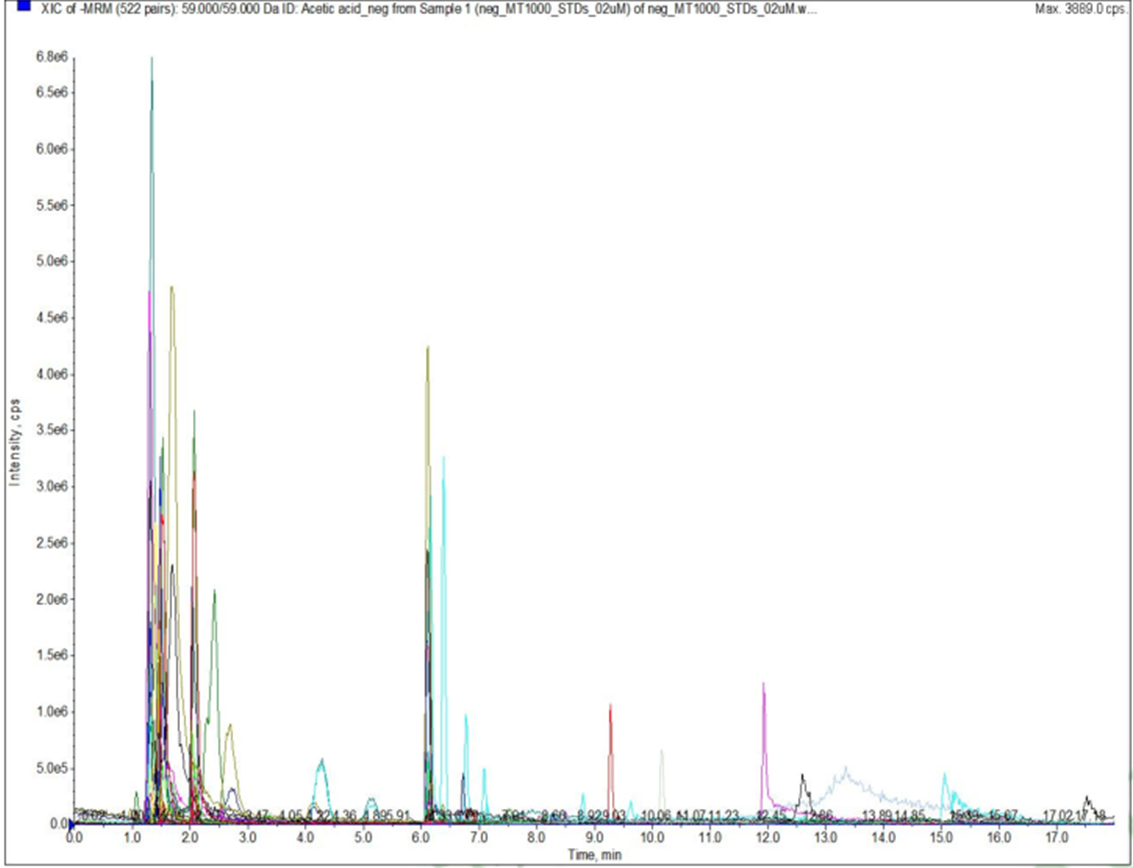


Figure S2. Extracted ion chromatogram (XIC) of mixed samples in negative ion mode.

The abscissa is the retention time (RT) of metabolites detection, and the ordinate is the ion current intensity of the detection (CPS, count per second).


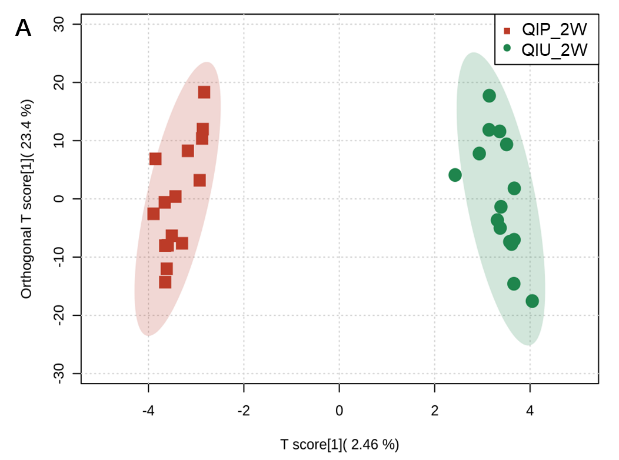

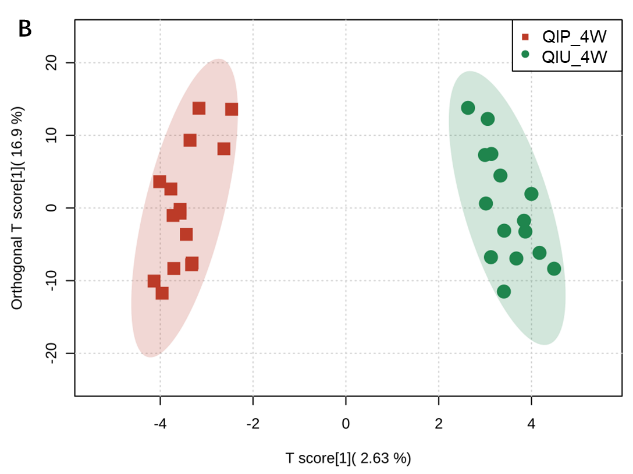


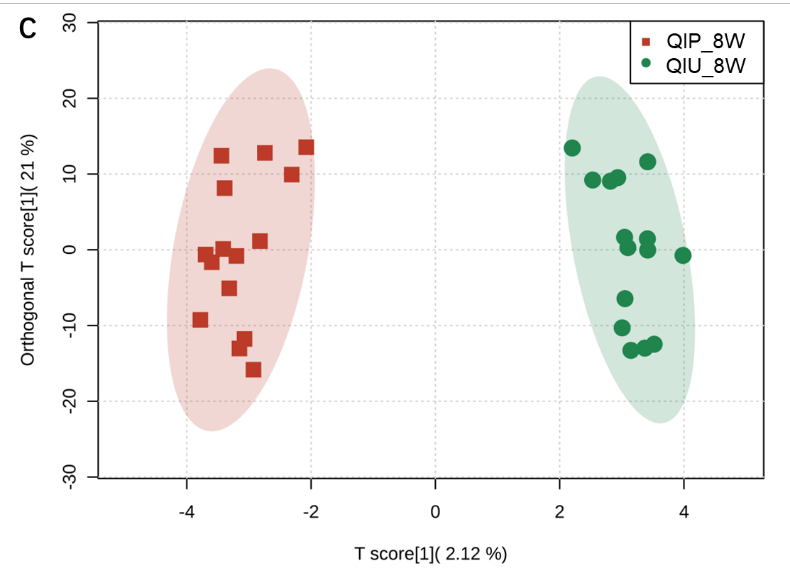


Figure S3. OPLS-DA score plots for the validation cohort

A: QIP_2W vs. QIU_2W; B: QIP_4W vs. QIU_4W; C: QIP_8W vs. QIU_8W


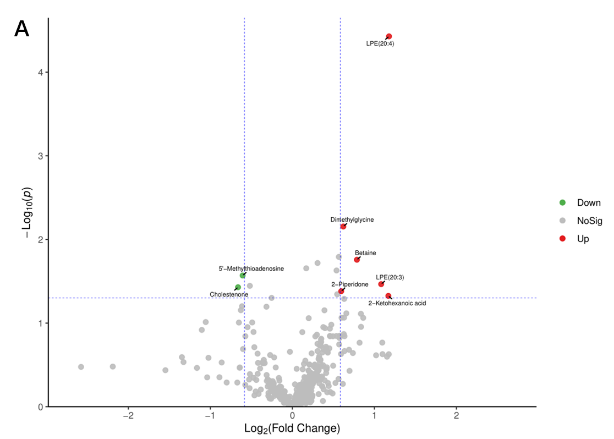

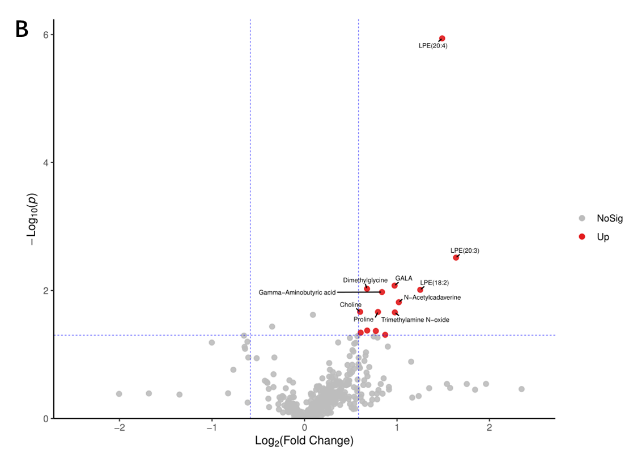


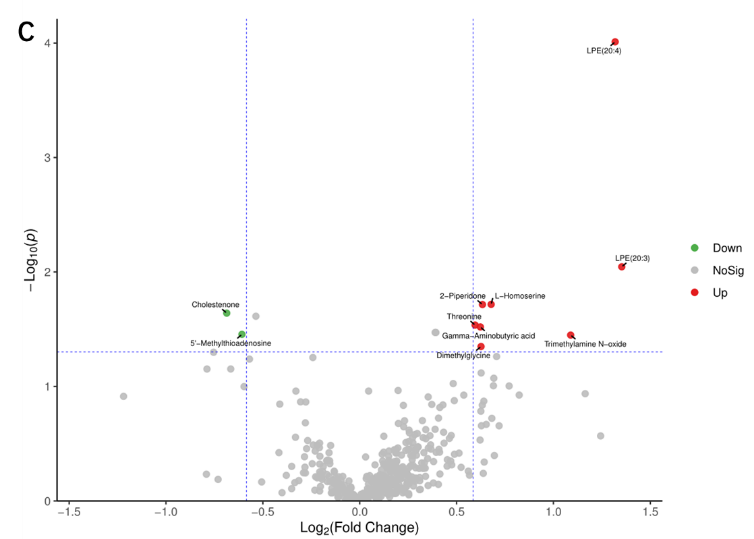


Figure S4. Volcano maps for the validation cohort

A: QIP_2W vs. QIU_2W; B: QIP_4W vs. QIU_4W; C: QIP_8W vs. QIU_8W


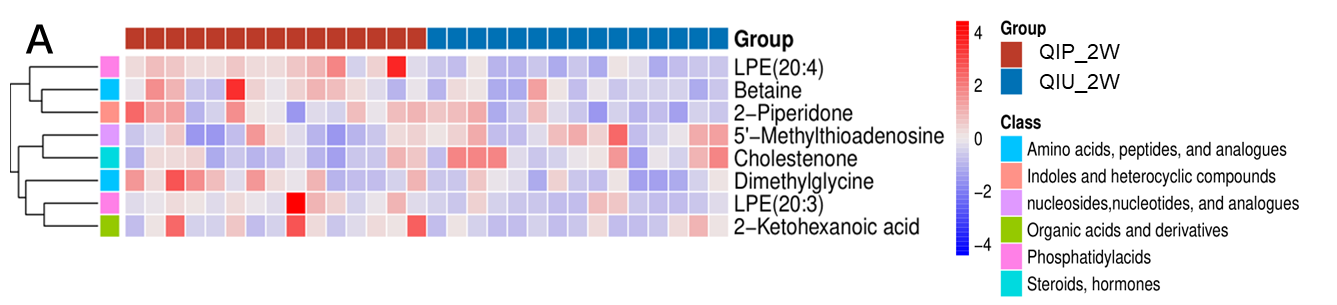


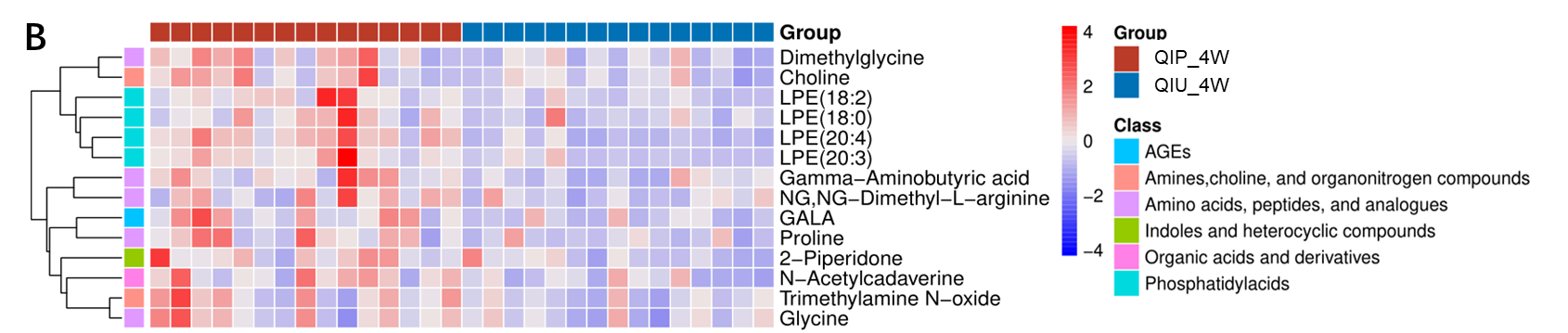


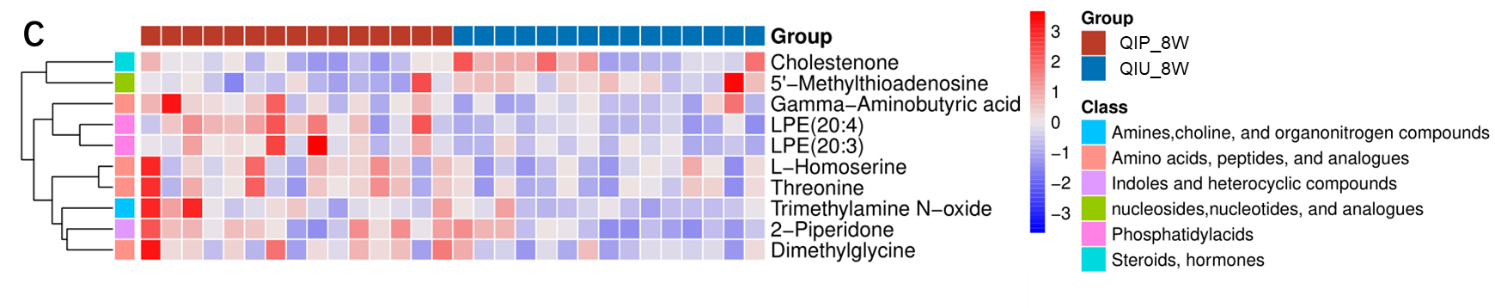


Figure S5. Heat maps for the validation cohort

A: QIP_2W vs. QIU_2W; B: QIP_4W vs. QIU_4W; C: QIP_8W vs. QIU_8W
